# A meta-analysis on the effectiveness of serotype O foot-and-mouth disease vaccines

**DOI:** 10.1038/s41598-025-99518-3

**Published:** 2025-05-02

**Authors:** Jiao Jiao, Haihua Yang, Zhenqi Liang, Yanhui Pan, Jiaxin Yang, Wenli Zhang, Peng Wu

**Affiliations:** 1 College of Life Sciences, Shihezi University, Shihezi, China; 2 Ministry of Education Key Laboratory of Xinjiang Phytomedicine Resource Utilization, Shihezi, China; 3 Xinjiang Production and Construction Corps Key Laboratory of Oasis Town and Mountain-Basin System Ecology, Shihezi, China; 4 State Key Laboratory of Pathogen and Biosecurity, Shihezi, China

**Keywords:** Foot-and-mouth-disease, Vaccine, Meta-analysis, Method, Animal, Zoology, Diseases

## Abstract

Foot-and-mouth disease (FMD) is a highly contagious viral disease of domesticated animals that causes major economic losses globally. In this meta-analysis, 29 studies were evaluated using a random-effects model to analyze the efficacy of FMD vaccines. The quantifying heterogeneity between the groups was low (tau^2^ = 0.000, tau = 0.000, and I^2^ = 0.0% [0.0%; 24.6%]). The meta-analysis revealed that the inactivated vaccine provides the best protection among different vaccine types, with the following ranking from highest to lowest efficacy: inactivated vaccine > mRNA vaccine > *E. coli* vaccine > plant vaccine > recombinant virus vaccine > phage vaccine > synthesize vaccine > DNA vaccine > negative control. The findings revealed that the inactivated vaccine provides the best protection among the different types of vaccines. Based on these findings, we recommend using inactivated vaccines as controls in the development of novel vaccines, as they achieved the highest efficacy among all evaluated vaccine types.

## Introduction

Foot-and-mouth disease (FMD), one of the most contagious animal diseases, occurs in most parts of Africa, Asia, the Middle East, and South America^1^. More than 100 species of wild, laboratory, or domesticated animals have been infected with the FMD virus (FMDV) naturally or experimentally^2^. In endemic areas alone, the production losses and vaccination costs caused by FMD amount to US$6.5 to US$21 billion annually^3^.

Meta-analysis can be used to compare differences in the effects of various FMD vaccines to identify the most effective vaccines^4^. Network meta-analysis (NMA) is superior to the traditional paired meta-analysis method, as it evaluates the efficacy of interventions within a single framework, improves accuracy, compares intervention pairs that have never been directly compared in experiments, and provides the level of interventions according to their effectiveness^5^.

FMD is highly contagious, which hinders the performance of virus challenge studies, leading to a relative lack of data on virus challenges. Therefore, the data of virus challenge reported in the literature is very valuable^6^. As a step toward filling this research gap, this meta-analysis compared the effects of different vaccines. This study used Bayesian network meta-analysis to compare the protective effects of different FMD vaccines and identify the most effective vaccine.

## Materials and methods

### Search strategy

This study was conducted following the Preferred Reporting Items for Systematic Reviews and Meta-Analyses statement guidelines. The literature retrieved in this meta-analysis was collected by searching databases from their inception until August 2024 and evaluated by three researchers (PW, ZQL, and JJ). “King of Medical Literature” software was used to search for and delete duplicate files. The National Library of Medicine (Medline via PubMed) and Embase databases were searched using the keywords (FMDV AND vaccine) OR (FMDV AND protect). The China National Knowledge Infrastructure and Wanfang Data databases were searched for studies on FMDV vaccines using the keywords “FMDV,” “protect,” and “vaccine” using King of Medical Literature software.

### Inclusion and exclusion criteria

The following inclusion criteria were used: (1) the virus serotype must be type O. There are several reasons why FMDV serotype O is included. There is no cross protection between different serotypes of FMDV^7^. First, the FMDV serotype O serotype has the widest global distribution, covering multiple regions, including Asia, Africa, Europe, and the Americas, and as such, its harm to livestock far exceeds that of other serotypes^8,9^. Second, the structure of FMDV serotype O is the most unstable and easily degraded, and its vaccine immune effect is the worst^10,11^. Third, among FMD vaccines, O serum vaccine has enough data to meet the requirements of high-quality meta-analysis^12–14^. (2) the vaccine must be an FMD vaccine; and (3) the vaccine protective effects against FMDV must be evaluated and include challenge potency studies (direct potency studies, not only serology studies) with FMDV. The following exclusion criteria were used: (1) the carrier was not related to FMD; (2) the results did not provide the necessary basic data; and (3) the number of challenged animals was not recorded.

### Data extraction and summation

Two researchers conducted a preliminary screening by reading the titles and abstracts of the retrieved studies before reading the full text. They then made their selection according to the inclusion and exclusion criteria. JJ and ZQL extracted the data, and if they disagreed about any aspect of the extraction, PW made the final decision. The data extracted included the first author’s name, publication date, and total number of animals in the trials.

### Statistical analysis

R 4.3.0 language can be used to conduct Bayesian meta-analysis using JAGS 4.3.0 software. In R4.3.0, the “meta,” “grid,” and “net meta” libraries were used. The occurrence of zero events leading to bias was caused by the small number of animals involved. When the number of events was zero, the total number of events increased by 0.01. When all cases had events, 0.01 was subtracted from the total number of events. The random-effects model was used for meta-analysis to calculate the risk ratio along with a 95% confidence interval for dichotomous results^15^. The calculation of the random-effects model was actively performed using R software^16^. Where applicable, the results from the individual trials estimated were presented in a forest plot. The different FMD vaccine groups were compared in R4.3.0. Tau^2^ was used to quantify heterogeneity.

## Results

### Study identification

**Fig. 1:**
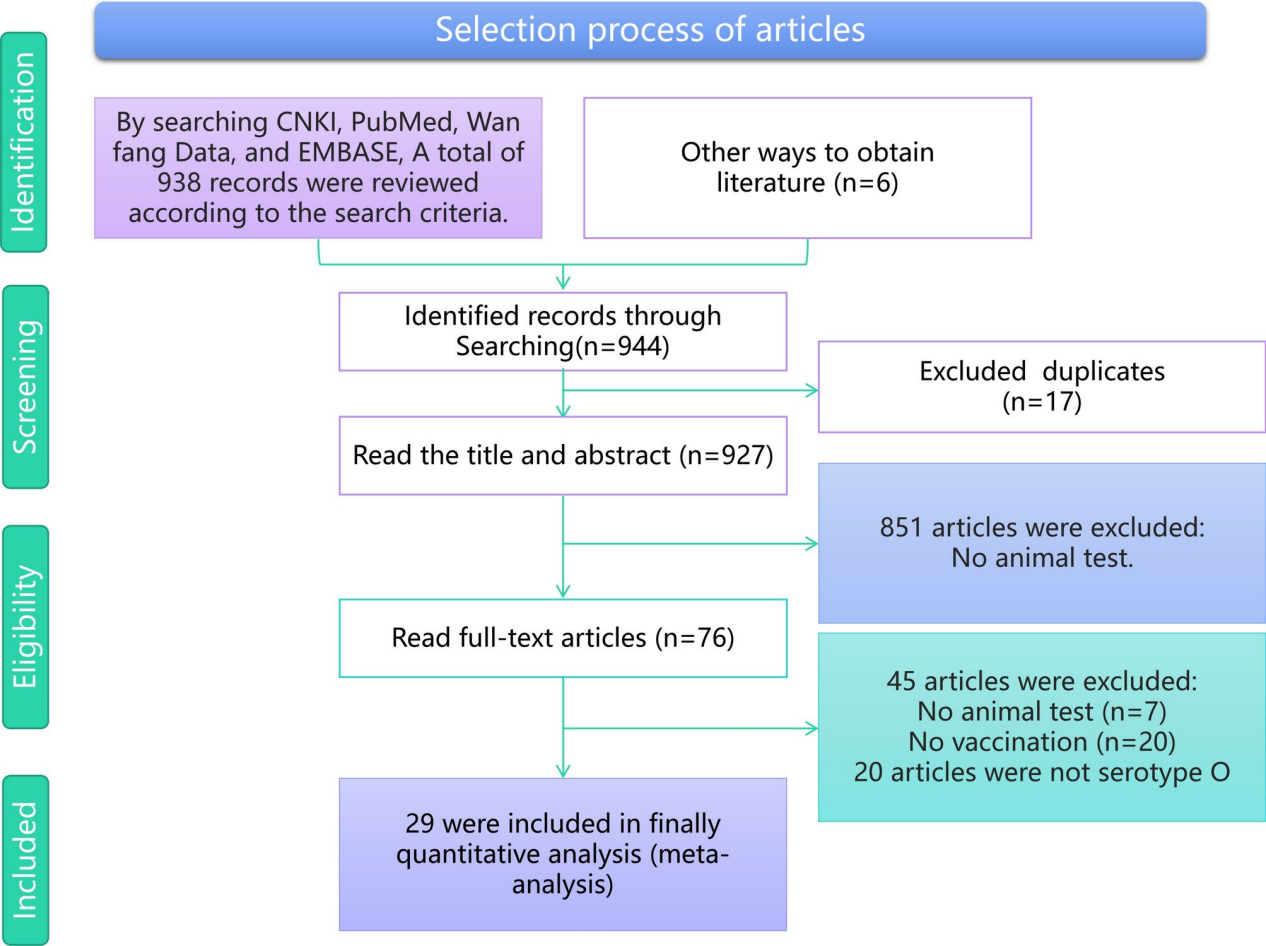
Flowchart of the article selection process.

Figure 1 presents the flowchart of the article selection process. Of the 944 articles screened, 76 were relevant for full-text review. These articles contained a total of 29 studies that underwent meta-analysis.

### Study characteristics

The selected articles included 29 studies focusing on FMD using the FMDV strain^17^. All studies evaluated the protective effects of FMD vaccines and performed virus challenge studies (Table 1).

**Table 1:** Protective effects of FMD vaccines^18–43^.

Number	Author	Year	Animal	Serotype	Viral infection serotype	Strain	Adjuvant	Infection dosage	Immunizing dose	Evaluation criteria	Experimental group	Number of protected animals	Total number of animals
1	Bingzhou Lu	2024	guinea pigs	None	O	O/Mya98/BY/2010	ISA 206 adjuvant	100 GPID_50_	None	Lesion	Contol group	0	5
2	Bingzhou Lu	2024	guinea pigs	O	O	O/Mya98/BY/2010	ISA 206 adjuvant	100 GPID_50_	100 µg	Lesion	*E. coli* vaccine	4	5
3	Bingzhou Lu	2024	guinea pigs	O	O	O/Mya98/BY/2010	ISA 206 adjuvant	100 GPID_50_	None	Lesion	Inactivated vaccine	5	5
4	Junjun Shao A	2024	pigs	O	O	O/Mya98/BY/2010	ISA 206 VG	1000 PID_50_	400 µg	Lesion	*E. coli* vaccine	5	5
5	Junjun Shao A	2024	pigs	O	O	O/Mya98/BY/2010	ISA 206 VG	1000 PID_50_	200 µg	Lesion	*E. coli* vaccine	5	5
6	Junjun Shao A	2024	pigs	O	O	O/Mya98/BY/2010	ISA 206 VG	1000 PID_50_	100 µg	Lesion	*E. coli* vaccine	5	5
7	Junjun Shao A	2024	pigs	O	O	O/Mya98/BY/2010	ISA 206 VG	1000 PID_50_	50 µg	Lesion	*E. coli* vaccine	5	5
8	Junjun Shao A	2024	pigs	None	O	O/Mya98/BY/2010	ISA 206 VG	1000 PID_50_	None	Lesion	Contol group	0	3
9	Junjun Shao C	2024	pigs	O	O	O/Mya98/BY/2010	ISA 206 VG	1000 PID_50_	400 µg	Lesion	*E. coli* vaccine	10	10
10	Junjun Shao C	2024	pigs	O	O	O/Mya98/BY/2010	ISA 206 VG	1000 PID_50_	400 µg	Lesion	*E. coli* vaccine	10	10
11	Junjun Shao C	2024	pigs	O	O	O/Mya98/BY/2010	ISA 206 VG	1000 PID_50_	400 µg	Lesion	*E. coli* vaccine	10	10
12	Junjun Shao C	2024	pigs	None	O	O/Mya98/BY/2010	ISA 206 VG	1000 PID_50_	None	Lesion	Contol group	0	3
13	DONG Jin-Jie	2022	guinea pigs	O	O	O/MYA98/BY/2010	None	100 GPID_50_	None	Lesion	Inactivated vaccine	4	4
14	DONG Jin-Jie	2022	guinea pigs	O	O	O/MYA98/BY/2010	None	100 GPID_50_	None	Lesion	Inactivated vaccine	3	4
15	DONG Jin-Jie	2022	guinea pigs	None	O	O/MYA98/BY/2010	LNP	100 GPID_50_	None	Lesion	Contol group	0	4
16	DONG Jin-Jie	2022	guinea pigs	None	O	O/MYA98/BY/2010	None	100 GPID_50_	None	Lesion	Contol group	0	4
17	DONG Jin-Jie	2022	guinea pigs	O	O	O/MYA98/BY/2010	None	50 GPID_50_	None	Lesion	Inactivated vaccine	4	4
18	DONG Jin-Jie	2022	guinea pigs	O	O	O/MYA98/BY/2010	None	50 GPID_50_	None	lesion	Inactivated vaccine	4	4
19	DONG Jin-Jie	2022	guinea pigs	None	O	O/MYA98/BY/2010	LNP	50 GPID_50_	None	Lesion	Contol group	0	4
20	DONG Jin-Jie	2022	guinea pigs	None	O	O/MYA98/BY/2010	None	50 GPID_50_	None	Lesion	Contol group	0	4
21	Xiaoni Shi	2022	guinea pigs	O	O	O/China99	SNA	100 ID_50_	50 µg	Lesion	*E. coli* vaccine	5	6
22	Xiaoni Shi	2022	guinea pigs	O	O	O/China99	ISA-206	100 ID_50_	50 µg	Lesion	*E. coli* vaccine	5	6
23	Xiaoni Shi	2022	guinea pigs	None	O	O/China99	None	100 ID_50_	50 µg	Lesion	Contol group	0	6
24	Giselle Rangel	2021	pigs	O	O	O/UKG/11/01	ISA 50V2	5000 TCID_50_	2 mg	Lesion	Recombinant virus	1	3
25	Giselle Rangel	2021	pigs	O	O	O/UKG/11/01	ISA 50V2	5000 TCID_50_	0.2 mg	Lesion	Recombinant virus	0	3
26	Giselle Rangel	2021	pigs	None	O	O/UKG/11/01	ISA 50V2	5000 TCID_50_	None	lesion	Contol group	0	2
27	Hyundong Jo	2021	pigs	None	O	O/SKR/BE/2017	10% Al(OH)3	100,000 TCID_50_	None	Lesion	Contol group	0	3
28	Hyundong Jo	2021	pigs	O	O	O/SKR/BE/2017	10% Al(OH)3	100,000 TCID_50_	0.015 mg	Lesion	*E. coli* vaccine	2	3
29	Hyundong Jo	2021	pigs	O	O	O/SKR/BE/2017	10% Al(OH)3	100,000 TCID_50_	0.15 mg	Lesion	*E. coli* vaccine	5	5
30	Hyundong Jo	2021	mice	None	O	O/VET/2013	None	100 LD_50_	None	Alive	Contol group	0	5
31	Hyundong Jo	2021	mice	O	O	O/VET/2013	None	100 LD_50_	10 μg	Alive	*E. coli* vaccine	4	5
32	Hyundong Jo	2021	mice	None	O	O/VET/2013	None	100,000 TCID_50_	None	Alive	Contol group	0	10
33	Hyundong Jo	2021	mice	O	O	O/VET/2013	10% Al(OH)3	100,000 TCID_50_	None	Alive	*E. coli* vaccine	4	10
34	Hyundong Jo	2021	mice	O	O	O/VET/2013	rpHSP70-AD	100,000 TCID_50_	None	Alive	*E. coli* vaccine	6	10
35	Yunqi Yang	2021	guinea pigs	None	O	O/BY/CHA/2010	None	100 ID_50_	100 μg	Lesion	Contol group	0	4
36	Yunqi Yang	2021	guinea pigs	None	O	O/BY/CHA/2010	None	100 ID_50_	100 μg	Lesion	Contol group	0	4
37	Yunqi Yang	2021	guinea pigs	O	O	O/BY/CHA/2010	PLGA	100 ID_50_	100 μg	lesion	DNA vaccine	1	4
38	Yunqi Yang	2021	guinea pigs	O	O	O/BY/CHA/2010	PLGA IL-2	100 ID_50_	100 μg	Lesion	DNA vaccine	2	4
39	Yunqi Yang	2021	guinea pigs	O	O	O/BY/CHA/2010	PLGA IL-18	100 ID_50_	100 μg	Lesion	DNA vaccine	3	4
40	Yunqi Yang	2021	guinea pigs	O	O	O/BY/CHA/2010	GM-CSF	100 ID_50_	100 μg	Lesion	DNA vaccine	2	4
41	Yunqi Yang	2021	guinea pigs	O	O	O/BY/CHA/2010	None	100 ID_50_	100 μg	Lesion	Inactivated vaccine	4	4
42	Gyeongmin Lee	2020	mice	None	O	O/SKR/Boeun/2017	None	200 LD_50_	None	Alive	Contol group	0	10
43	Gyeongmin Lee	2020	mice	O	O	O/SKR/Boeun/2017	Montanide ISA 201	200 LD_50_	1 µg	Alive	Inactivated vaccine	10	10
44	Gyeongmin Lee	2020	mice	O	O	O/SKR/Boeun/2017	Montanide ISA 201	200 LD_50_	0.25 µg	Alive	Inactivated vaccine	10	10
45	Gyeongmin Lee	2020	mice	O	O	O/SKR/Boeun/2017	Montanide ISA 201	200 LD_50_	0.063 µg	Alive	Inactivated vaccine	8	10
46	Gyeongmin Lee	2020	mice	O	O	O/SKR/Boeun/2017	Montanide ISA 201	200 LD_50_	0.016 µg	Alive	Inactivated vaccine	8	10
47	Gyeongmin Lee	2020	mice	O	O	O/SKR/Boeun/2017	Montanide ISA 201	200 LD_50_	1 µg	Alive	Inactivated vaccine	10	10
48	Gyeongmin Lee	2020	mice	O	O	O/SKR/Boeun/2017	Montanide ISA 201	200 LD_50_	0.1 µg	Alive	Inactivated vaccine	10	10
49	Rodrigo Cañas-Arranz	2019	pigs	O	O	O/UKG 11/01	None	100 TCID_50_	2 mg	Lesion	Synthesize vaccine	4	5
50	Rodrigo Cañas-Arranz	2019	pigs	O	O	O/UKG 11/01	None	100 TCID_50_	0.5 mg	Lesion	Synthesize vaccine	4	5
51	Rodrigo Cañas-Arranz	2019	pigs	None	O	O/UKG 11/01	None	100 TCID_50_	PBS	Lesion	Contol group	0	2
52	Guoqiang Wang	2018	pigs	O	O	O/Mya98/BY/2010	ISA 50V2	1000 ID_50_	1 mL	Lesion	Inactivated vaccine	5	5
53	Guoqiang Wang	2018	pigs	O	O	O/Mya98/BY/2010	ISA 50V2	1000 ID_50_	100 μg	Lesion	*E. coli* vaccine	2	5
54	Guoqiang Wang	2018	pigs	None	O	O/Mya98/BY/2010	ISA 50V2	1000 ID_50_	None	Lesion	Contol group	0	3
55	Hai Xu	2017	pigs	O	O	O/MYA98	ISA206	100 BID_50_	None	Lesion	Phage vaccine vaccine vaccine	4	5
56	Hai Xu	2017	pigs	O	O	O/MYA98	ISA206	100 BID_50_	0.5 mg	Lesion	Synthesize vaccine	3	5
57	Hai Xu	2017	pigs	O	O	O/MYA98	ISA206	100 BID_50_	None	Lesion	Inactivated vaccine	5	5
58	Hai Xu	2017	pigs	None	O	O/MYA98	ISA206	100 BID_50_	None	Lesion	Contol group	0	2
59	Haitao Li	2015	guinea pigs	O	O	O/CHA/99	ISA-206	100 GPID_50_	30 μg	Lesion	Recombinant virus	3	4
60	Haitao Li	2015	guinea pigs	O	O	O/CHA/99	ISA-206	100 GPID_50_	None	Lesion	Inactivated vaccine	3	4
61	Haitao Li	2015	guinea pigs	None	O	O/CHA/99	None	100 GPID_50_	None	Lesion	Contol group	0	4
62	Yanmei Dong	2015	pigs	None	O	O/OZK	None	1000 ID_50_	None	Lesion	Contol group	0	5
63	Yanmei Dong	2015	pigs	O	O	O/OZK	None	1000 ID_50_	10 µg	Lesion	Inactivated vaccine	4	5
64	Yanmei Dong	2015	pigs	O	O	O/OZK	None	1000 ID_50_	10 µg	Lesion	Phage vaccine vaccine vaccine	1	5
65	Yanmei Dong	2015	pigs	O	O	O/OZK	None	1000 ID_50_	10 µg	Lesion	*E. coli* vaccine	3	5
66	Yanmei Dong	2015	guinea pigs	None	O	O/OZK	None	1000 ID_50_	None	Lesion	Contol group	0	20
67	Yanmei Dong	2015	guinea pigs	O	O	O/OZK	None	1000 ID_50_	0.2 mg	Lesion	Inactivated vaccine	16	20
68	Yanmei Dong	2015	guinea pigs	O	O	O/OZK	None	1000 ID_50_	0.2 mg	Lesion	Phage vaccine vaccine vaccine	5	20
69	Yanmei Dong	2015	guinea pigs	O	O	O/OZK	None	1000 ID_50_	0.2 mg	Lesion	*E. coli* vaccine	13	20
70	Xiaohu Wang	2010	guinea pigs	O	O	O/China99	None	1000 LD_50_	600 μg	Lesion	DNA vaccine	3	7
71	Xiaohu Wang	2010	guinea pigs	O	O	O/China99	None	1000 LD_50_	600 μg	Lesion	DNA vaccine	3	7
72	Xiaohu Wang	2010	guinea pigs	O	O	O/China99	None	1000 LD_50_	600 μg	Lesion	DNA vaccine	2	7
73	Xiaohu Wang	2010	guinea pigs	O	O	O/China99	None	1000 LD_50_	600 μg	Lesion	DNA vaccine	2	7
74	Xiaohu Wang	2010	guinea pigs	O	O	O/China99	None	1000 LD_50_	None	Lesion	Inactivated vaccine	4	4
75	Xiaohu Wang	2010	guinea pigs	None	O	O/China99	None	1000 LD_50_	None	Lesion	Contol group	0	4
76	M HEMA	2009	cattle	None	O	O1 BFS 1860	ISA 206 VG	10,000 ID_50_	10 μg	Lesion	Contol group	0	4
77	M HEMA	2009	cattle	O	O	O1 BFS 1860	ISA 206 VG	10,000 ID_50_	10 μg	Lesion	Inactivated vaccine	4	4
78	M HEMA	2009	cattle	O	O	O1 BFS 1860	ISA 206 VG	10,000 ID_50_	10 μg	Lesion	Inactivated vaccine	4	4
79	Zhaojun Ren	2008	mice	O	O	O/Yub-1	None	1,000,000 TCID_50_	200 CFU	Alive	Phage vaccine vaccine vaccine	22	22
80	Zhaojun Ren	2008	mice	O	O	O/Yub-1	None	1,000,000 TCID_50_	200 CFU	Alive	Phage vaccine vaccine vaccine	11	20
81	Zhaojun Ren	2008	mice	O	O	O/Yub-1	None	1,000,000 TCID_50_	200 CFU	Alive	Phage vaccine vaccine vaccine	0	10
82	Zhaojun Ren	2008	mice	O	O	O/Yub-1	None	1,000,000 TCID_50_	200 CFU	Alive	Phage vaccine vaccine vaccine	18	24
83	Zhaojun Ren	2008	mice	O	O	O/Yub-1	None	1,000,000 TCID_50_	200 CFU	Alive	Phage vaccine vaccine vaccine	8	21
84	Zhaojun Ren	2008	mice	O	O	O/Yub-1	None	1,000,000 TCID_50_	200 CFU	Alive	Phage vaccine vaccine vaccine	0	6
85	Zhaojun Ren	2008	mice	None	O	O/Yub-1	None	1,000,000 TCID_50_	200 CFU	Alive	Contol group	0	12
86	Zhaojun Ren	2008	mice	O	O	O/Yub-1	None	1,000,000 TCID_50_	200 CFU	Alive	Inactivated vaccine	6	6
87	Zhaojun Ren	2008	mice	None	O	O/Yub-1	None	1,000,000 TCID_50_	200 CFU	Alive	Contol group	0	10
88	ChungDa Yang	2007	pigs	O	O	O/Taiwan/97	ISA206	100,000 TCID_50_	5 mg	Lesion	Recombinant virus	3	3
89	ChungDa Yang	2007	pigs	O	O	O/Taiwan/97	ISA206	100,000 TCID_50_	1 mg	Lesion	Recombinant virus	3	3
90	ChungDa Yang	2007	pigs	O	O	O/Taiwan/97	ISA-206	100,000 TCID_50_	0.5 mg	Lesion	Recombinant virus	3	3
91	ChungDa Yang	2007	pigs	None	O	O/Taiwan/97	ISA206	100,000 TCID_50_	None	Lesion	Contol group	0	2
92	ChungDa Yang	2007	pigs	None	O	O/Taiwan/97	None	100,000 TCID_50_	None	Lesion	Contol group	0	2
93	Pan Li	2006	guinea pigs	None	O	O/China/99	Freund’s adjuvant	100 ID_50_	100 mg	Lesion	Contol group	0	10
94	Pan Li	2006	guinea pigs	O	O	O/China/99	Freund’s adjuvant	100 ID_50_	100 mg	Lesion	Phage vaccine	6	10
95	Houhui Song	2005	pigs	O	O	None	None	1,000,000 TCID_50_	0.1 mg	Lesion	Phage vaccine	8	10
96	Houhui Song	2005	pigs	None	O	None	None	1,000,000 TCID_50_	None	Lesion	Contol group	0	10
97	Houhui Song	2005	mice	None	O	None	None	100,000 SMLD_50_	None	Lesion	Contol group	0	20
98	Houhui Song	2005	mice	O	O	None	None	100,000 SMLD_50_	2–4 μg	Lesion	Phage vaccine	59	90
99	Guangjin Li	2004	guinea pigs	O	O	None	None	100 ID_50_	100 µg	Lesion	*E. coli* vaccine	6	6
100	Guangjin Li	2004	guinea pigs	O	O	None	None	100 ID_50_	100 µg	Lesion	*E. coli* vaccine	6	6
101	Guangjin Li	2004	guinea pigs	None	O	None	None	100 ID_50_	None	Lesion	Contol group	0	21
102	Guangjin Li	2004	pigs	O	O	None	None	100 ID_50_	1.6 mg	Lesion	*E. coli* vaccine	5	5
103	Guangjin Li	2004	pigs	O	O	None	None	100 ID_50_	1.6 mg	Lesion	*E. coli* vaccine	5	5
104	Guangjin Li	2004	pigs	None	O	None	None	100 ID_50_	None	Lesion	Contol group	0	4
105	Ligang Wu	2003	guinea pigs	O	O	None	7% aluminum hydroxide in mineral oil	GPID_50_	0.6 mg	Lesion	Recombinant virus	6	6
106	Ligang Wu	2003	guinea pigs	O	O	None	7% aluminum hydroxide in mineral oil	GPID_50_	0.6 mg	Lesion	Recombinant virus	4	6
107	Ligang Wu	2003	guinea pigs	O	O	None	7% aluminum hydroxide in mineral oil	GPID_50_	0.6 mg	Lesion	Recombinant virus	3	4
108	Ligang Wu	2003	guinea pigs	None	O	None	7% aluminum hydroxide in mineral oil	GPID_50_	0.6 mg	Lesion	Contol group	0	6
109	Ligang Wu	2003	pigs	O	O	None	7% aluminum hydroxide in mineral oil	20 MID	3 mg	Lesion	Recombinant virus	3	3
110	Ligang Wu	2003	pigs	None	O	None	7% aluminum hydroxide in mineral oil	20 MID	3 mg	Lesion	Contol group	0	3
111	Ligang Wu	2003	guinea pigs	None	O	None	7% aluminum hydroxide in mineral oil	50 GPID_50_	0.5 mg	Lesion	Contol group	0	4
112	Ligang Wu	2003	guinea pigs	O	O	None	7% aluminum hydroxide in mineral oil	50 GPID_50_	0.5 mg	Lesion	Recombinant virus	4	4
113	Ligang Wu	2003	guinea pigs	O	O	None	7% aluminum hydroxide in mineral oil	100 GPID_50_	0.5 mg	Lesion	Recombinant virus	6	6
114	Ligang Wu	2003	guinea pigs	O	O	None	7% aluminum hydroxide in mineral oil	150 GPID_50_	0.5 mg	Lesion	Recombinant virus	6	6
115	Chunjian Tian	2003	mice	O	O	O/GD-10	None	None	None	Lesion	Inactivated vaccine	6	6
116	Chunjian Tian	2003	mice	O	O	O/GD-10	None	None	10000000 CFU	Lesion	Phage vaccine vaccine vaccine	3	5
117	Chunjian Tian	2003	mice	O	O	O/GD-10	None	None	10000,000 CFU	Lesion	Phage vaccine vaccine vaccine	5	5
118	Chunjian Tian	2003	mice	None	O	O/GD-10	None	None	None	Lesion	Contol group	0	5
119	Ping Qian	2004	pigs	O	O	F29	None	20 MID	100000 TCID_50_	Lesion	Recombinant virus	0	5
120	Ping Qian	2004	pigs	O	O	F29	Oil adjuvant	20 MID	100000 TCID_50_	Lesion	Inactivated vaccine	5	5
121	Ping Qian	2004	pigs	None	O	F29	None	20 MID	100000 TCID_50_	Lesion	Contol group	0	5
122	Jingyun Ma	2004	guinea pigs	O	O	None	None	None	20 μg	Lesion	*E. coli* vaccine	2	4
123	Jingyun Ma	2004	guinea pigs	None	O	None	None	None	None	Lesion	Contol group	0	4
124	Jeng-Hwan Wang	2003	pigs	None	O	O/Taiwan/97	Freund’s adjuvant	100,000 TCID_50_	None	Lesion	Contol group	0	2
125	Jeng-Hwan Wang	2003	pigs	O	O	O/Taiwan/97	Freund’s adjuvant	100,000 TCID_50_	3 mg	Lesion	*E. coli* vaccine	11	12
126	C. CARRILLO	2001	mice	None	O	O1C	None	10,000 SMLD_50_	15 to 20 mg	Absence of viremia	Contol group	0	18
127	C. CARRILLO	2001	mice	O	O	O1C	None	10000 SMLD_50_	15 to 20 mg	Absence of viremia	Phage vaccine	41	49
128	EWC chan	2001	pigs	O	O	O1K HK	None	50 LD_50_	2 mg	Lesion	*E. coli* vaccine	5	5
129	EWC chan	2001	pigs	O	O	O1K HK	None	50 LD_50_	0.5 mg	Lesion	*E. coli* vaccine	2	5
130	EWC chan	2001	pigs	None	O	O1K HK	None	50 LD_50_	0.5 mg	Lesion	Contol group	0	5
131	EWC chan	2001	pigs	O	O	O1K HK	None	50 LD_50_	10000000 TCID_50_	Lesion	Inactivated vaccine	5	5
132	EWC chan	2001	pigs	None	O	O1K HK	None	50 LD_50_	None	Lesion	Contol group	0	5
133	A Wigdorovitz	1999	mice	None	O	O1C	None	10,000 SMLD_50_	None	Absence of viremia	Contol group	0	46
134	A Wigdorovitz	1999	mice	O	O	O1C	None	10,000 SMLD_50_	None	Absence of viremia	Phage vaccine	38	50
135	C. CARRILLO	1998	mice	None	O	O1C	None	10,000 SMLD_50_	None	Absence of viremia	Contol group	0	12
136	C. CARRILLO	1998	mice	O	O	O1C	None	10,000 SMLD_50_	None	Absence of viremia	Phage vaccine	14	14

### Meta-analysis

**Fig. 2:**
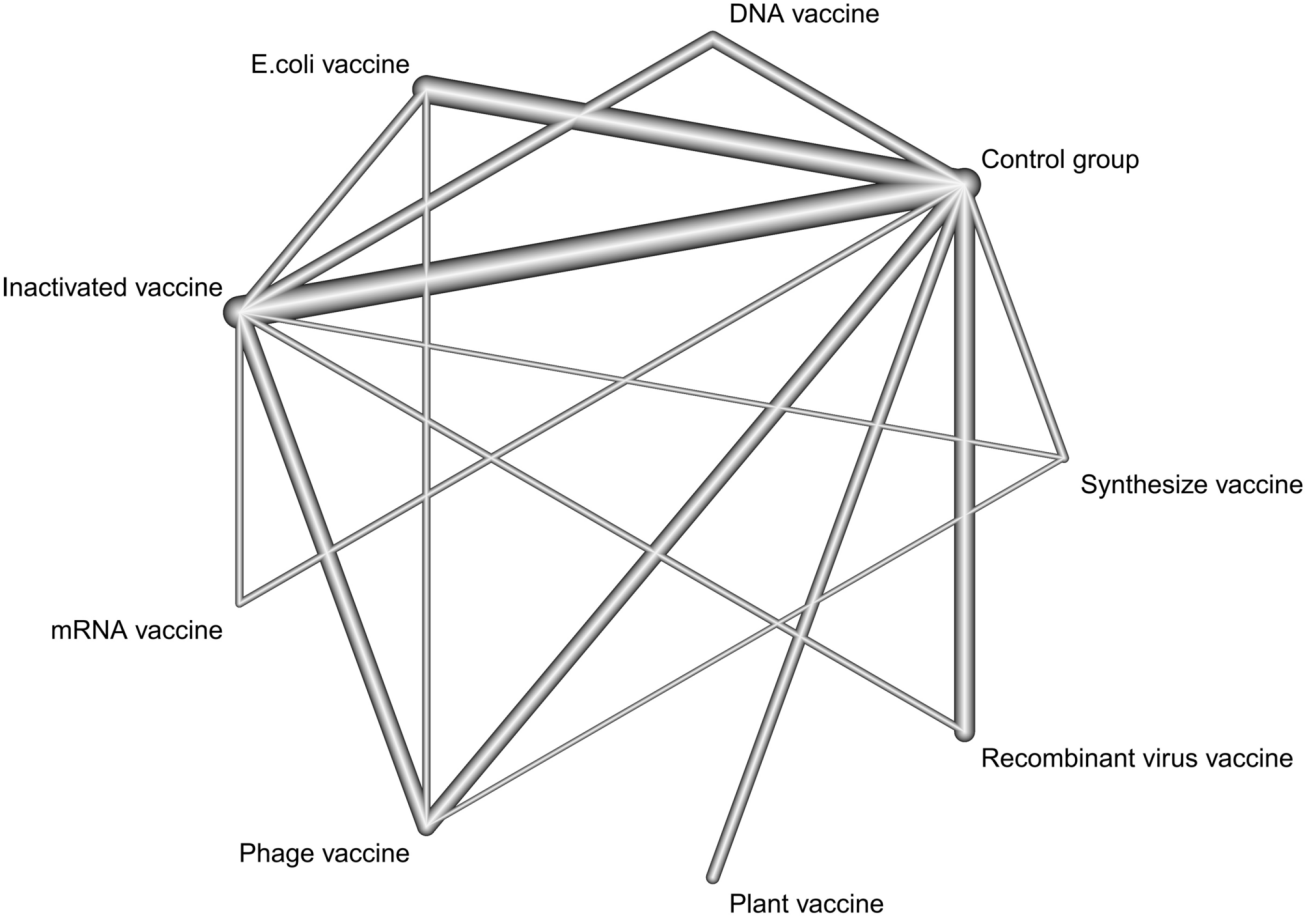
Network of FMD vaccines.

The network of FMD vaccines is presented in Fig. 2. The results indicated that the number of comparisons made with blank groups, inactivated vaccines, recombinant virus vaccines, and *Escherichia coli* vaccines was the largest. All groups were directly compared with the blank control group.

**Fig. 3:**
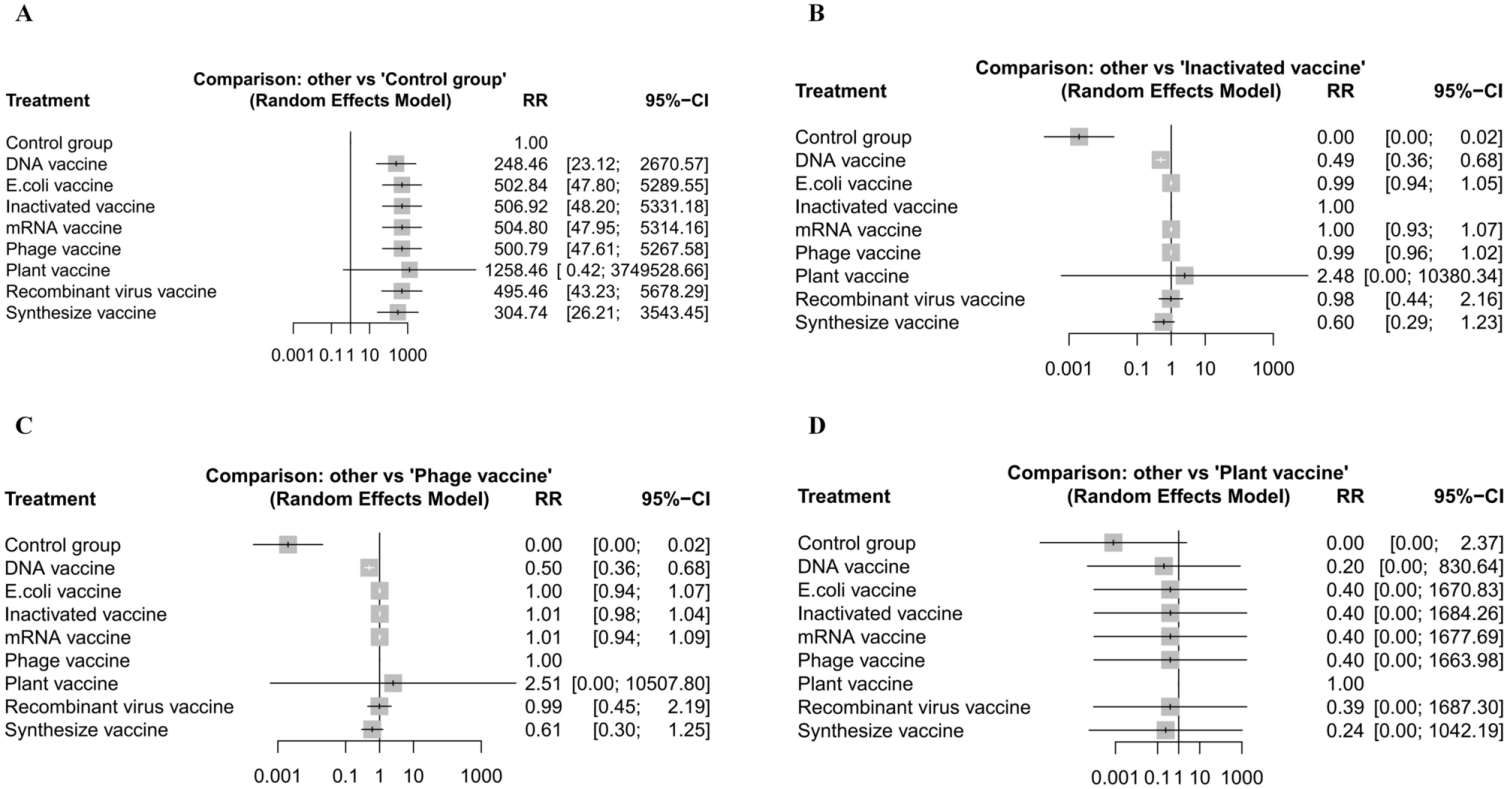
Forest plot.

Furthermore, the quantifying heterogeneity between the groups was low (tau^2^ = 0.000, tau = 0.000, and I^2^ = 0.0% [0.0%; 24.6%]) (Fig. 3).

**Fig. 4:**
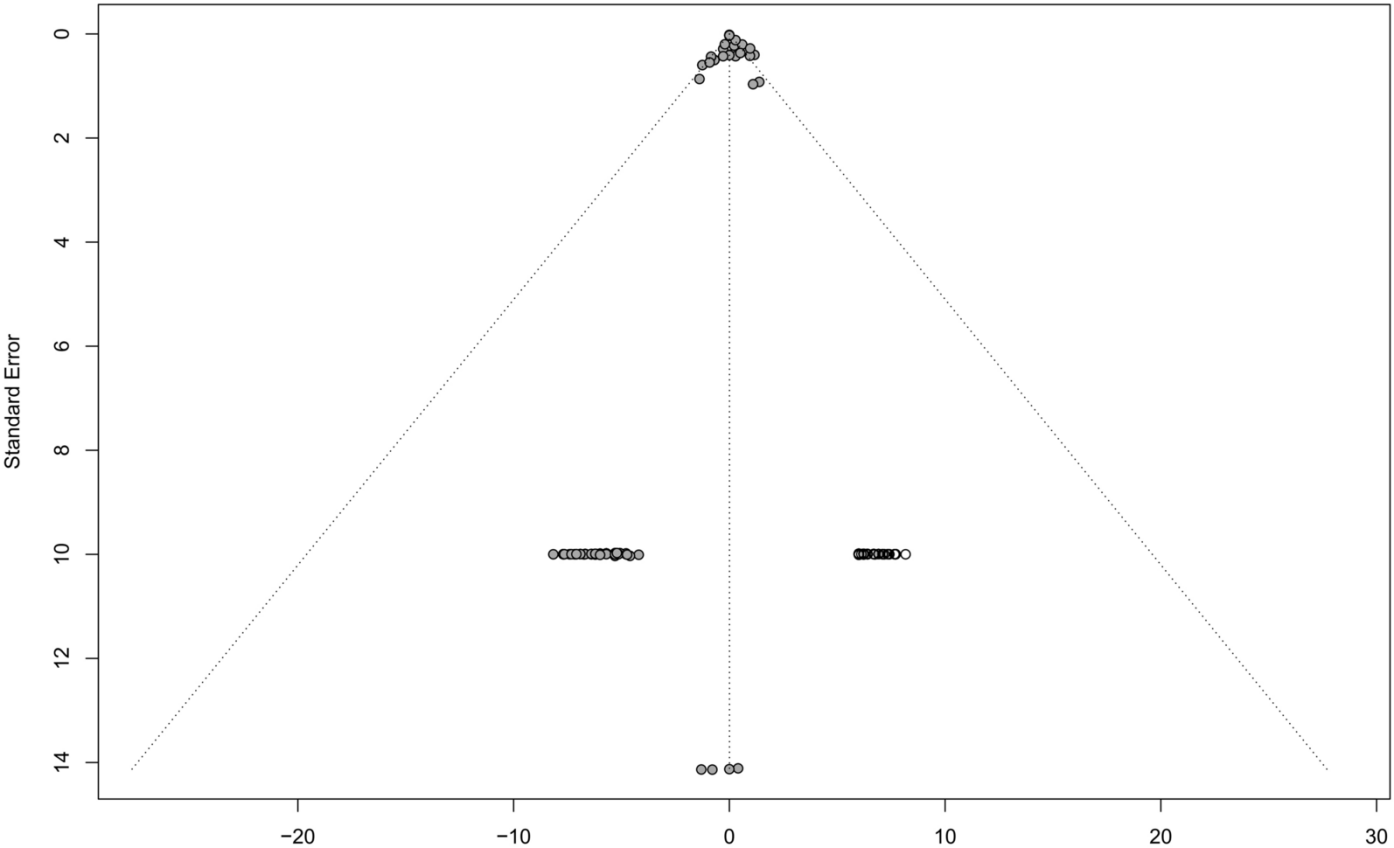
Funnel plot.

A funnel plot was developed for visual investigation of possible small-study effects. Overall, the plot resembled a funnel chart. Funnel plot analysis revealed that the bias between the groups was within an acceptable range (Fig. 4). The result of heterogeneity testing was small, with tau^2^ = 0.000.

The results of the meta-analysis indicated that FMD vaccines produced through different methods provide different degrees of protection, with the following ranking from highest to lowest efficacy: inactivated vaccine > mRNA vaccine > *E. coli* vaccine > plant vaccine > recombinant virus vaccine > phage vaccine > synthesize vaccine > DNA vaccine > negative control (Table 2).

**Table 2:** Ranking of FMD vaccines based on their efficacy.

	P-score (random)
Inactivated vaccine	0.7277
mRNA vaccine	0.6774
*E. coli* vaccine	0.6559
Plant vaccine	0.6457
Recombinant virus vaccine	0.6381
Phage vaccine	0.6123
Synthesize vaccine	0.3238
DNA vaccine	0.2142
Negative control	0.005

**Fig. 5:**
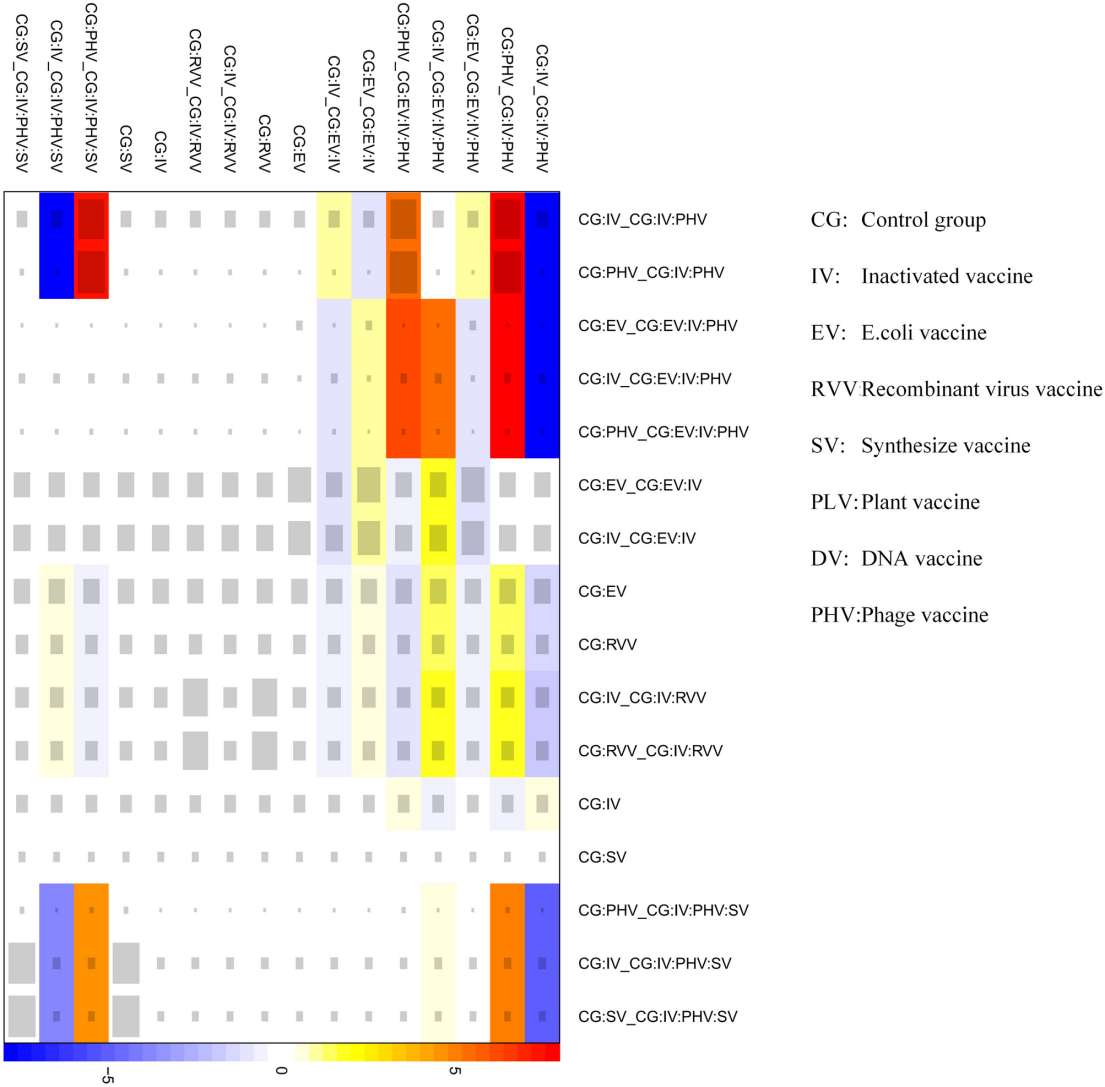
Heat map.

Figure 5 shows that research has primarily focused on inactivated, phage, and peptide vaccines and that relatively little research has investigated plant-derived vaccines. Among all vaccines, inactivated FMD vaccines are the most extensively used.

## Discussion

Currently, the primary method of preventing animals from being infected with FMD virus is through vaccination. With the continuous progress of biotechnology, new vaccines are constantly being developed and undergoing continuous research and further development. The performance of FMD vaccines is inconsistent, and the identification of the most effective vaccine remains controversial. This study compared the data on all vaccines with known efficacy, particularly vaccines that have not undergone direct comparison, to determine their value and guide their follow-up in vaccine development. The inactivated vaccine (0.728) has the highest score, indicating its effectiveness and reliability. It is a traditional vaccine type, in which the virus is chemically inactivated but retains its ability to induce an immune response. Its high score suggests widespread use, proven efficacy, and safety in controlling FMD outbreaks^44^. However, it may require cold chain storage and multiple doses for sustained immunity^45^. mRNA vaccines (0.6774) represent a modern approach, leveraging genetic technology to stimulate immune responses. Although mRNA vaccines scored slightly less than inactivated vaccines, they offer advantages such as rapid development and scalability. Their lower score might reflect challenges in stability, delivery, or limited long-term data in FMD applications^46^. *E. coli* vaccines (0.6559) use *E. coli* as a vector to express FMD antigens. Their scores indicate moderate effectiveness, likely due to their cost-effectiveness and ease of production^47,48^. However, they may face limitations in antigen presentation or immune response robustness compared with other methods. DNA vaccines (0.2142) have the lowest score, indicating limited success in FMD applications. Although they offer simplicity in design, they often struggle with low immunogenicity, delivery efficiency, and regulatory hurdles. Inactivated vaccines remain the most effective option for FMD, while mRNA and E. coli vaccines show promise as modern alternatives^49,50^. Plant and recombinant virus vaccines offer innovative approaches but face practical challenges^51,52^. Phage, synthesized, and DNA vaccines are currently less effective, highlighting the need for further research and development^53,54^.

FMD is highly contagious, which hinders the performance of virus challenge studies, leading to a relative lack of data on virus challenges. As a step toward filling this research gap, this meta-analysis compared the effects of different vaccines. Under Bayesian mediation analysis, the inference is straightforward and exact, which makes it appealing for studies with small samples^55^, and this is one of the most widely used methods^56,57^. The advantages of meta-analysis include the provision of a comprehensive retrieval strategy and qualification criteria for retrieval research. Nevertheless, the use of meta-analysis in this study posed several limitations. The possibility of publishing studies with undesired results is low, which could lead to higher vaccine protection data^58^. First, FMDV is highly infectious, which limits the number of animals that can be used for experiments. Accordingly, having insufficient data, such as in studies related to new vaccines, increases the deviation of the results. Second, there may be bias in the funnel plot used for the visual (and fully subjective) investigation of possible small-study effects^59^. Third, a source of heterogeneity may be related to the dose of the vaccine; some vaccines are dose-dependent, only exerting fully protective effects at high doses^60^.

Only FMDV serotype O was included for several reasons. Although there are some data on FMDV serotype A, more research is needed to meet the requirements of meta-analysis^61,62^. For these reasons, it has the most important value for the study of the FMD vaccine. NMA can contribute to resolving controversies, reducing the reliance on laboratory animals, avoiding duplication of work, and guiding future research directions. NMA helps researchers study important and previously unanswerable questions, which has contributed to the rapid increase in the number of studies using NMA in the biomedical literature^4^. To our knowledge, our study is the first to use NMA to investigate FMD vaccines, making our study design innovative and our findings significant.

## Conclusions

In this meta-analysis, 29 studies were evaluated to analyze the efficacy of FMD vaccines. The findings revealed that the inactivated vaccine provides the best protection among the different types of vaccines. Based on these findings, we recommend using inactivated vaccines as controls in the development of novel vaccines, as they achieved the highest efficacy among all evaluated vaccine types.

## Data Availability

All data generated or analysed during this study are included in this published article.

## References

[1] Hammond, J. M., Maulidi, B. & Henning, N. Targeted FMD vaccines for Eastern Africa: The AgResults foot and mouth disease vaccine challenge project. *Viruses* **13**, 1830 (2021). 34578411 10.3390/v13091830 PMC8472200

[2] Weaver, G. V., Domenech, J., Thiermann, A. R. & Karesh, W. B. Foot and mouth disease: A look from the wild side. *J. Wildl. Dis.* **49**, 759–785 (2013). 24502706 10.7589/2012-11-276

[3] Stenfeldt, C. et al. Virulence beneath the fleece; A tale of foot-and-mouth disease virus pathogenesis in sheep. *PLoS ONE* **14**, e0227061 (2019). 31891626 10.1371/journal.pone.0227061 PMC6938329

[4] Watt, J. & Del Giovane, C. Network meta-analysis. *Methods Mol. Biol.* **2345**, 187–201 (2022). 34550592 10.1007/978-1-0716-1566-9_12

[5] Seitidis, G., Nikolakopoulos, S., Hennessy, E. A., Tanner-Smith, E. E. & Mavridis, D. Network meta-analysis techniques for synthesizing prevention science evidence. *Prev. Sci.* **23**, 415–424 (2022). 34387806 10.1007/s11121-021-01289-6

[6] Sanghi, D. K. & Tiwle, R. A detail comprehensive review on vaccines. *Int. J. Res. Dev. Pharmacy Life Sci.* **3**, 887–895 (2014).

[7] Momtaz, S., Towheed, S. T., Ali, R., Ullah, H. & Hossain, M. A. Molecular epidemiology of foot-and-mouth disease virus serotypes circulating in Bangladesh. *Microbiol. Res. J. Int.* **158**, 82–94 (2012).

[8] An, Q. et al. Global foot-and-mouth disease risk assessment based on multiple spatial analysis and ecological niche model. *Vet. Q.* **45**, 1–11 (2025). 39838825 10.1080/01652176.2025.2454482 PMC11755741

[9] Verma, A. K., Kumar, A., Mahima, & Sahzad,. Epidemiology and diagnosis of foot-and-mouth disease: A review. *Indian J. Animal Sci.* **82**, 543–551 (2012).

[10] Pacheco, J. M. & Mason, P. W. Evaluation of infectivity and transmission of different Asian foot-and-mouth disease viruses in swine. *J. Vet. Sci.* **11**, 133–142 (2010). 20458154 10.4142/jvs.2010.11.2.133 PMC2873813

[11] Juan, et al. Evaluation of infectivity, virulence and transmission of FDMV field strains of serotypes O and A isolated in 2010 from outbreaks in the Republic of Korea. *PLoS ONE* **11**, 1–21 (2016). 10.1371/journal.pone.0146445 PMC4703371 26735130

[12] Tsai, C. P., Pan, C. H., Liu, M. Y., Lin, Y. L. & Yang, P. C. Molecular epidemiological studies on foot-and-mouth disease type O Taiwan viruses from the 1997 epidemic. *Vet. Microbiol.* **74**, 207–216 (2000). 10808089 10.1016/s0378-1135(00)00182-6

[13] Tang, H., Liu, X. S., Fang, Y. Z., Pan, L. & Zhang, Y. G. Geometrical study on FMDV genome based on Z-curve. *J. Anim. Vet. Adv.* **11**, 2630–2640 (2012).

[14] Abubakar, M., Arshed, M. J., Ali, Q. & Hussain, M. Spatial trend of Foot and Mouth Disease virus (FMDV) serotypes in cattle and buffaloes, Pakistan. *Virol. Sin.* **27**, 320–323 (2012). 23055008 10.1007/s12250-012-3271-8 PMC8218023

[15] Kanters, S. Fixed- and random-effects models. *Methods Mol. Biol.* **2345**, 41–65 (2022). 34550583 10.1007/978-1-0716-1566-9_3

[16] Nestler, S. & Erdfelder, E. Random effects multinomial processing tree models: A maximum likelihood approach. *Psychometrika* **88**, 809–829 (2023). 37247167 10.1007/s11336-023-09921-w PMC10444666

[17] Hajam, I. A. et al. Co-administration of flagellin augments immune responses to inactivated foot-and-mouth disease virus (FMDV) antigen. *Res. Vet. Sci.* **95**, 936–941 (2013). 23941960 10.1016/j.rvsc.2013.07.021

[18] Lee, G. et al. Vaccine strain of O/ME-SA/Ind-2001e of foot-and-mouth disease virus provides high immunogenicity and broad antigenic coverage. *Antiviral Res.* **182**, 104920 (2020). 32828822 10.1016/j.antiviral.2020.104920

[19] Hema, M., Chandran, D., Nagendrakumar, S. B., Madhanmohan, M. & Srinivasan, V. A. Construction of an infectious cDNA clone of foot-and-mouth disease virus type O 1 BFS 1860 and its use in the preparation of candidate vaccine. *J. Biosci.* **34**, 45–58 (2009). 19430118 10.1007/s12038-009-0008-4

[20] Lu, B. et al. A ferritin-based nanoparticle displaying a neutralizing epitope for foot-and-mouth disease virus (FMDV) confers partial protection in guinea pigs. *BMC Vet. Res.* **20**, 301 (2024). 38971791 10.1186/s12917-024-04159-9 PMC11227194

[21] Shao, J., Liu, W., Gao, S., Chang, H. & Guo, H. A recombinant multi-epitope trivalent vaccine for foot-and-mouth disease virus serotype O in pigs. *Virology* **596**, 110103 (2024). 38781710 10.1016/j.virol.2024.110103

[22] Shi, X. et al. Development and efficacy evaluation of a novel nano-emulsion adjuvant for a foot-and-mouth disease virus-like particles vaccine based on squalane. *Nanomaterials (Basel)* **12**, 3934 (2022). 36432220 10.3390/nano12223934 PMC9698784

[23] Rangel, G. et al. Chimeric RHDV virus-like particles displaying foot-and-mouth disease virus epitopes elicit neutralizing antibodies and confer partial protection in pigs. *Vaccines (Basel)* **9**, 470 (2021). 34066934 10.3390/vaccines9050470 PMC8148555

[24] Jo, H. et al. The HSP70-fused foot-and-mouth disease epitope elicits cellular and humoral immunity and drives broad-spectrum protective efficacy. *NPJ Vaccines* **6**, 42 (2021). 33772029 10.1038/s41541-021-00304-9 PMC7998017

[25] Yang, Y. et al. Enhanced immunogenicity of foot and mouth disease DNA vaccine delivered by PLGA nanoparticles combined with cytokine adjuvants. *Res. Vet. Sci.* **136**, 89–96 (2021). 33592449 10.1016/j.rvsc.2021.02.010

[26] Cañas-Arranz, R. et al. A single dose of dendrimer B(2)T peptide vaccine partially protects pigs against foot-and-mouth disease virus infection. *Vaccines (Basel)* **8**, 19 (2020). 31936706 10.3390/vaccines8010019 PMC7157199

[27] Xu, H. et al. Immunogenicity of T7 bacteriophage nanoparticles displaying G-H loop of foot-and-mouth disease virus (FMDV). *Vet. Microbiol.* **205**, 46–52 (2017). 28622860 10.1016/j.vetmic.2017.04.023

[28] Li, H. et al. Novel chimeric foot-and-mouth disease virus-like particles harboring serotype O VP1 protect guinea pigs against challenge. *Vet. Microbiol.* **183**, 92–96 (2016). 26790940 10.1016/j.vetmic.2015.12.004

[29] Dong, Y. M., Zhang, G. G., Huang, X. J., Chen, L. & Chen, H. T. Promising MS2 mediated virus-like particle vaccine against foot-and-mouth disease. *Antiviral Res.* **117**, 39–43 (2015). 25676866 10.1016/j.antiviral.2015.01.005

[30] Wang, X. H., Jin, Y. Z. & Fan, X. B. Construction and Immunogenicity of DNA Vaccine Plasmid Expressing VP1 and VP4 Genes of Foot-and-Mouth Disease Virus. *Chin. J. Biol.* **23**, 168–171+175 (2010).

[31] Ren, Z. J. et al. Orally delivered foot-and-mouth disease virus capsid protomer vaccine displayed on T4 bacteriophage surface: 100% protection from potency challenge in mice. *Vaccine* **26**, 1471–1481 (2008). 18289743 10.1016/j.vaccine.2007.12.053

[32] Yang, C. et al. Induction of protective immunity in swine by recombinant bamboo mosaic virus expressing foot-and-mouth disease virus epitopes. *BMC Biotechnol.* **7**, 62 (2007). 17900346 10.1186/1472-6750-7-62 PMC2117009

[33] Li, P., Yong-Guang, Z., Yong-Lu, W., Bao-Qin, W. & Qing-Ge, X. Protective immune response of guinea pigs against challenge with foot and mouth disease virus by immunization with foliar extracts from transgenic tomato plants expressing the FMDV structural protein VP1. *Wei sheng wu xue bao Acta microbiologica Sinica* **46** (2006). 17172031

[34] Song, H. H. et al. A novel mucosal vaccine against foot-and-mouth disease virus induces protection in mice and swine. *Biotechnol. Lett.* **27**, 1669–1674 (2005). 16247672 10.1007/s10529-005-2727-4

[35] Li, G. J. et al. Comparison of immune responses against foot-and-mouth disease virus induced by fusion proteins using the swine IgG heavy chain constant region or beta-galactosidase as a carrier of immunogenic epitopes. *Virology* **328**, 274–281 (2004). 15464847 10.1016/j.virol.2004.07.025

[36] Wang, J. H. et al. Induction of immunity in swine by purified recombinant VP1 of foot-and-mouth disease virus. *Vaccine* **21**, 3721–3729 (2003). 12922103 10.1016/s0264-410x(03)00363-3

[37] Wu, L. et al. Expression of foot-and-mouth disease virus epitopes in tobacco by a tobacco mosaic virus-based vector. *Vaccine* **21**, 4390–4398 (2003). 14505922 10.1016/s0264-410x(03)00428-6

[38] Qian, P., Li, X. M., Jin, M. L., Peng, G. Q. & Chen, H. C. An approach to a FMD vaccine based on genetic engineered attenuated pseudorabies virus: One experiment using VP1 gene alone generates an antibody responds on FMD and pseudorabies in swine. *Vaccine* **22**, 2129–2136 (2004). 15149769 10.1016/j.vaccine.2003.12.005

[39] Carrillo, C. et al. Induction of a virus-specific antibody response to foot and mouth disease virus using the structural protein VP1 expressed in transgenic potato plants. *Viral Immunol.* **14**, 49–57 (2001). 11270596 10.1089/08828240151061383

[40] Chan, E. W. et al. An immunoglobulin G based chimeric protein induced foot-and-mouth disease specific immune response in swine. *Vaccine* **19**, 538–546 (2000). 11027819 10.1016/s0264-410x(00)00186-9

[41] Wigdorovitz, A. et al. Induction of a protective antibody response to foot and mouth disease virus in mice following oral or parenteral immunization with alfalfa transgenic plants expressing the viral structural protein VP1. *Virology* **255**, 347–353 (1999). 10069960 10.1006/viro.1998.9590

[42] Carrillo, C., Wigdorovitz, A., Oliveros, J. C., Zamorano, P. I. & Borca, M. V. Protective immune response to foot-and-mouth disease virus with VP1 expressed in transgenic plants. *J. Virol.* **72**, 1688–1690 (1998). 9445079 10.1128/jvi.72.2.1688-1690.1998 PMC124657

[43] Jing-Yun, M. A., Feng, C., Yong-Chang, C., Qing-Feng, Z. & Ying-Zuo, B. I. Prokaryotic expression of VP1 gene of foot-and-mouth disease virus and detection of expression product immunogenicity. *Chin. J. Vet. Sci. Technol.* **34**, 17–20 (2004).

[44] Wu, P. et al. Layered double hydroxide nanoparticles as an adjuvant for inactivated foot-and-mouth disease vaccine in pigs. *BMC Vet. Res.* **16**, 474 (2020). 33276787 10.1186/s12917-020-02689-6 PMC7716589

[45] Ranjan, R., Biswal, J. K., Sahoo, P. K., Tripathy, J. P. & Singh, R. P. Diagnostic application of formalin fixed archived tissues for detection of foot-and-mouth disease. *J. Virol. Methods* **318**, 1–6 (2023). 10.1016/j.jviromet.2023.114754 37230193

[46] Borrego, B. et al. Delivery of synthetic RNA can enhance the immunogenicity of vaccines 1 against foot-and-mouth disease virus (FMDV) in mice. *Vaccine* **31**, 4375–4381 (2013). 23859841 10.1016/j.vaccine.2013.07.008

[47] Kim, T. H. & Wijerathna, S. M. FMDV Multi-VP1e can induces protection against lethal FMDV challenge 1, 1–2 (2017).

[48] Liu, C. et al. Soluble FMDV VP1 proteins fused with calreticulin expressed in Escherichia coli under the assist of trigger factor16 (Tf16) formed into high immunogenic polymers. *Int. J. Biol. Macromol. Struct. Funct. Interact.* **15**, 1532–1540 (2020). 10.1016/j.ijbiomac.2019.11.130 31739054

[49] Cenikli, D. A comparison of production, efficacy, and safety of mRNA and conventional vaccines. *Int. J. High School Res.* **4**, 1–22 (2022).

[50] Lee, W. S. et al. Randomized trial of same- versus opposite-arm coadministration of inactivated influenza and SARS-CoV-2 mRNA vaccines. *JCI Insight* **10**, 1–11 (2025). 10.1172/jci.insight.187075 PMC11949032 39786918

[51] Geisbert, T. W. Recombinant vesicular stomatitis virus-based vaccines against Ebola and Marburg virus infections. *J. Infect. Dis.* **204**, S1075 (2011). 21987744 10.1093/infdis/jir349 PMC3218670

[52] Thanavala, Y., Huang, Z. & Mason, H. S. Plant-derived vaccines: a look back at the highlights and a view to the challenges on the road ahead. *Expert Rev. Vaccines* **5**(2), 249–260 (2006). 16608424 10.1586/14760584.5.2.249

[53] Chirico, F., Silva, J. A. T. D., Tsigaris, P. & Sharun, K. Safety & effectiveness of COVID-19 vaccines: A narrative review. *Indian J. Med. Res.* **155**, 91–104 (2022). 35859436 10.4103/ijmr.IJMR_474_21 PMC9552389

[54] Head, T., Rao, V. B. & Black, L. W. REVIEW Structure and assembly of bacteriophage. **1**, 69–110 (1977).

[55] Yuan, Y. & MacKinnon, D. P. Bayesian mediation analysis. *Psychol. Methods* **14**, 301–322 (2009). 19968395 10.1037/a0016972 PMC2885293

[56] Yarnell, C. J., Granton, J. T. & Tomlinson, G. Bayesian analysis in critical care medicine. *Am. J. Respir. Crit Care Med.* **201**, 396–398 (2020). 31899649 10.1164/rccm.201910-2019ED PMC7049930

[57] Kruschke, J. K. Bayesian analysis reporting guidelines. *Nat. Hum. Behav.* **5**, 1282–1291 (2021). 34400814 10.1038/s41562-021-01177-7 PMC8526359

[58] Zybert, A., Tarczyński, K. & Sieczkowska, H. Meta-analysis of the effect of chilling on selected attributes of fresh pork. *J. Food Process Preserv.* **43**, 1–9 (2019).

[59] Philip, S. & Marston, L. How to read a funnel plot in a meta-analysis. *BMJ (Clin. Res. Ed.)* **351**, 1–3 (2015). 10.1136/bmj.h4718 26377337

[60] Jiao, J. & Wu, P. A meta-analysis: the efficacy and effectiveness of polypeptide vaccines protect pigs from foot and mouth disease. *Sci. Rep.* **12**, 21868 (2022). 36536158 10.1038/s41598-022-26462-x PMC9763257

[61] Brehm, K. E., Kumar, N., Thulke, H. H. & Haas, B. High potency vaccines induce protection against heterologous challenge with foot-and-mouth disease virus. *Vaccine* **26**, 1681–1687 (2008). 18313814 10.1016/j.vaccine.2008.01.038

[62] Xie, Y., Chang, H., Li, Z. & Zhang, Y. Adenovirus-vectored capsid proteins of the serotype a foot-and-mouth disease virus protect guinea pigs against challenge. *Front Microbiol.* **11**, 1449 (2020). 32733405 10.3389/fmicb.2020.01449 PMC7363769

